# Quantifying the Association Between Obesity and In-Hospital Outcomes in Adult Scoliosis Surgery: A Nationwide Inpatient Sample Analysis (2016–2021)

**DOI:** 10.3390/jcm15176511

**Published:** 2026-08-23

**Authors:** Oded Rabau, Ahmad Essa, Yossi Smorgick, Yoram Anekstein, Jonathan Persitz, Anan Shtewe, Amr Mansour, Saleem Samara, Mohammad Haj Yahya, Eyal Behrbalk

**Affiliations:** 1 Spine Unit, Department of Orthopedic Surgery, Shamir (Assaf Harofeh) Medical Center, Zerifin 60930, Israelnuritan@zahav.net.il (Y.A.); 2 Faculty of Medicine, Tel Aviv University, Tel Aviv 6997801, Israel; 3 Hand Program, Divisions of Orthopaedic Surgery and Plastic, Reconstructive & Aesthetic Surgery, University Health Network, Department of Surgery, Temerty Faculty of Medicine, University of Toronto, 200 Elizabeth St, Toronto, ON M5G 2C4, Canada; 4 Department of Orthopaedic Surgery, Chaim Sheba Medical Center (Tel-Hashomer), Ramat Gan 52621, Israel; 5 Department of Orthopedics, Hillel Yaffe Medical Center, Hadera 3820302, Israel; amrmansour2011@gmail.com (A.M.); dr.saleem.a.s@gmail.com (S.S.); mohammadqhy@gmail.com (M.H.Y.); dr.behrbalk@gmail.com (E.B.); 6 Rappaport Faculty of Medicine, Technion University Hospital (Israel Institute of Technology), Haifa 3525433, Israel

**Keywords:** adult scoliosis, obesity, in-hospital complications, retrospective study, National Inpatient Sample

## Abstract

**Background/Objectives**: To quantify the adjusted association of obesity with in-hospital outcomes in adult scoliosis hospitalizations involving spine deformity corrective surgery. **Methods**: A retrospective cohort study was conducted using the National Inpatient Sample (2016–2021), including adult hospitalizations for scoliosis undergoing corrective spine surgery. Obesity was the primary exposure. The primary outcome was a composite of in-hospital complications. Secondary outcomes included individual complications, length of stay, total hospital charges, and discharge disposition. Weighted multivariable regression models adjusted for patient- and hospital-level factors were used. Sensitivity analysis was performed excluding severity adjustment to assess potential overadjustment, as the All Patient Refined Diagnosis Related Group (APR-DRG) severity score may partially reflect in-hospital complications. **Results**: Among 9323 unweighted hospitalizations (representing a national estimate of 46,610 weighted hospitalizations) included (16.6% obese), obesity was associated with a 22% increase in the odds of total in-hospital complications (Odds Ratio (OR) 1.22; 95% Confidence Interval (CI) 1.04–1.43). Among individual complication categories, obesity was associated with higher odds of renal complications (OR 1.69; 95% CI 1.29–2.21), whereas no significant differences were observed in pulmonary, cardiac, Venous Thromboembolism (VTE), or sepsis complications. Additionally, obesity was associated with increased odds of non-home discharge (OR 1.28; 95% CI 1.12–1.46). In sensitivity analysis, these associations were more pronounced, with additional complication categories reaching statistical significance. **Conclusions**: Obesity is associated with increased in-hospital morbidity in adult scoliosis surgery, particularly higher odds of renal complications and non-home discharge. These population-level findings help optimize preoperative risk communication and anticipate post-acute discharge resource needs.

## 1. Introduction

Obesity has become a global epidemic. As of 2022, an estimated 890 million adults worldwide met the World Health Organization’s definition of obesity [1]. Such a widespread demographic change presents a growing clinical challenge for spine surgeons, as obesity and its associated comorbidities are well-established risk factors for a broad spectrum of musculoskeletal disorders, including degenerative conditions of the spine [2,3,4,5]. Among these, adult spinal deformity (ASD) is particularly notable. In ASD, obesity has been identified as a significant, modifiable risk factor influencing disease onset, development, and progression [6,7,8,9].

While the general association between obesity and adverse outcomes following routine degenerative spine surgery is well established, the operative and physiological burden of adult scoliosis correction is fundamentally different. Deformity correction frequently involves long fusion constructs, extensive multidimensional correction, osteotomies, and a substantially higher baseline risk of acute organ-system complications compared to shorter-segment procedures. Despite the clinical significance of this relationship, reports on the association between obesity and in-hospital outcomes after surgical correction of scoliosis deformity remain conflicting. While several studies indicate that patients with obesity face increased risks [2,4,9,10,11,12,13,14], others show comparable complication rates to those of non-obese cohorts [15,16].

A major limitation of the existing literature is reliance on small, single-center cohorts [2,4,10,11,12,13,14,15,16]. Although prospective clinical registries provide critical granular data—such as measured continuous body mass index, operative variables, and longitudinal patient-reported outcomes—they predominantly capture shorter-segment procedures and often lack the statistical power to identify differences in lower-incidence, acute in-hospital organ-system complications specifically within the adult deformity population [11,14]. To address this gap, the present study utilized the National Inpatient Sample (NIS), the largest publicly available all-payer inpatient care database in the United States [17]. While administrative databases have inherent limitations, such as a lack of granular clinical data and long-term follow-up, they provide the substantial statistical power needed to detect and estimate relatively uncommon, acute in-hospital complications across a highly diverse national spectrum of institutions. Thus, this large-database analysis serves as a complementary epidemiological contribution to the complex and mixed literature.

The objective of this study was to evaluate the association between obesity and in-hospital outcomes among adults undergoing scoliosis-correction procedures in the United States.

## 2. Materials and Methods

### 2.1. Study Design and Data Source

A retrospective cohort study was conducted using the Healthcare Cost and Utilization Project (HCUP) National Inpatient Sample (NIS) database from 1 January 2016 to 31 December 2021. The NIS is the largest publicly available all-payer inpatient care database in the United States. Because the NIS contains fully de-identified, publicly available discharge-level data, institutional review board approval and individual informed consent were not required, and an ethical waiver was maintained. 

Additionally, this study followed the STROBE reporting guidelines [18]. A checklist is provided in Supplementary Table S2, which is attached in the Supplementary Materials.

### 2.2. Study Population

Eligible records were limited to hospitalizations from 1 January 2016 to 31 December 2021. To define the cohort, the following inclusion criteria were applied: (1) adults (≥18 years of age). (2) Scoliosis documented as the principal admission diagnosis (I10_DX1). (3) A qualifying spinal fusion surgery, listed as the primary procedure for scoliosis correction, with at least one complete, seven-character ICD-10-PCS scoliosis-correction procedure code.

To exclude acute emergencies and further isolate structural deformity correction from routine degenerative indications, the following exclusion criteria were applied: (1) Hospitalizations originating from the emergency department. (2) Records with secondary diagnoses of spinal tumors, vertebral fractures, spondylolisthesis, spondylolysis, or spinal stenosis.

**Figure 1:**
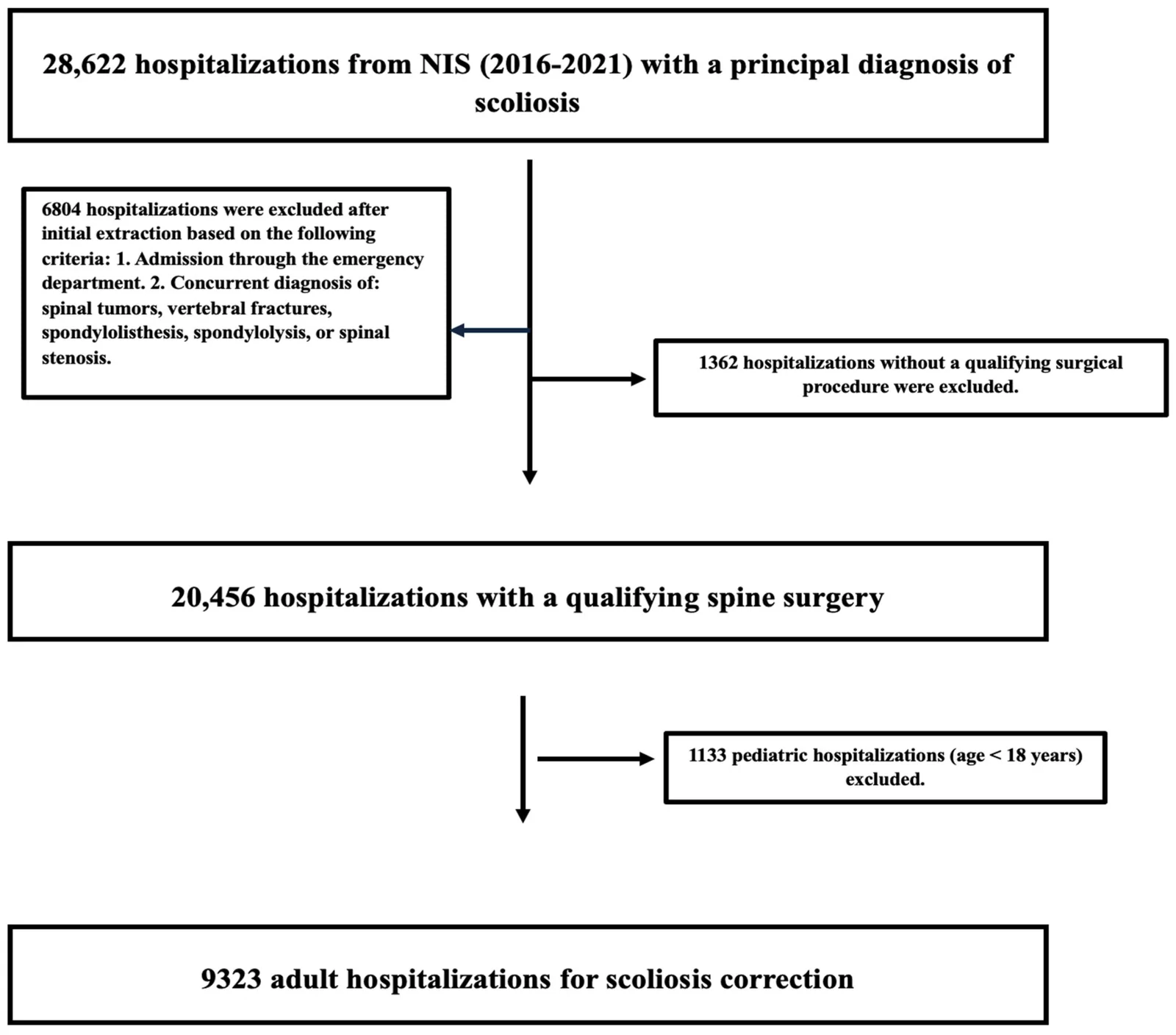
Cohort-selection flow diagram. All counts are unweighted hospitalizations.

The complete cohort algorithm and unweighted counts are shown in Figure 1, and all ICD-10 codes are detailed in Supplemental Table S1.

### 2.3. Variables and Outcomes

The primary exposure was binary administrative ICD-coded obesity. Although BMI-specific Z68 codes were initially queried during data extraction to stratify by obesity class, their high missingness and undercoding precluded reliable stratification, necessitating reliance on the binary codes.

The primary outcomes of the study were a composite of overall in-hospital complications and non-home discharge disposition. Individual organ-system complications, length of stay, and total hospital charges were evaluated as secondary, exploratory outcomes. Because diagnosis-specific present-on-admission (POA) indicators are not universally provided as a distinct variable in the NIS, adverse events were systematically distinguished from pre-existing comorbidities by utilizing ICD-10-CM codes specifically designated for intraoperative and postprocedural complications. The specific conditions comprising these categories are explicitly defined as follows: “Pulmonary Complications” includes pneumonia, acute respiratory distress syndrome (ARDS), acute respiratory failure, and other postprocedural respiratory disorders; “Cardiac Complications” encompasses acute myocardial infarction, cardiac arrest, cardiogenic shock, and other postprocedural heart failure; “Renal Complications” includes acute kidney failure and urinary tract infections; and “Venous Thromboembolism” encompasses both deep vein thrombosis and pulmonary embolism. These postprocedural codes are entirely distinct from the baseline comorbidity codes, as detailed in Supplemental Table S1.

### 2.4. Statistical Analysis

Descriptive statistics summarized all collected variables. Continuous variables were presented as means with standard deviations (SDs) and categorical variables as frequencies with percentages. Group comparisons were performed using weighted *t*-tests for continuous variables and χ^2^ or Fisher’s exact tests for categorical variables. Standardized mean differences (SMDs) were additionally calculated to assess the magnitude of baseline imbalances between the cohorts, with an absolute SMD greater than 0.10 considered indicative of a meaningful imbalance.

All analyses utilized survey-weighted generalized linear models accounting for strata, hospital-level clustering, and discharge weights in strict accordance with HCUP/NIS guidelines to generate nationally representative estimates. For categorical outcomes, survey-weighted logistic regression models were fitted, and results were reported as adjusted odds ratios (aORs) with 95% confidence intervals (CIs). Because length of stay was recorded as a nonnegative count of hospital days and exhibited overdispersion and right skewness, it was analyzed using a survey-weighted quasi-Poisson regression with a log link. Total hospital charges were analyzed using a survey-weighted Gamma regression with a log link because charges were continuous, positive, and right-skewed. Exponentiated coefficients were reported as adjusted incidence rate ratios (IRRs) for length of stay and adjusted mean ratios (aMRs) for total hospital charges, with corresponding 95% CIs.

To evaluate multiple outcomes hierarchically, overall in-hospital complications and non-home discharge were designated as primary endpoints, while specific individual organ-system complications were evaluated as secondary, exploratory outcomes intended to describe the anatomical drivers of the primary complication rate. In accordance with established epidemiological principles for observational research, formal multiplicity corrections were not applied to secondary endpoints, since these mathematical adjustments can significantly increase Type II error for biologically correlated outcomes; instead, the assessment relied on the magnitude of effect estimates and the precision of 95% confidence intervals. To assess whether the association between age and in-hospital complications differed by obesity status, a survey-weighted multivariable logistic regression model that included an obesity-by-age interaction was fitted, with age modeled using a natural cubic spline with four degrees of freedom. The overall interaction was evaluated using a design-adjusted joint Wald F-test for all obesity-by-spline terms. Adjusted predicted probabilities were calculated across the age range, adjusting for all other covariates, and were visualized graphically.

Two complementary sensitivity analyses were performed to evaluate the robustness of the primary models. First, the adjusted models were re-estimated after excluding APR-DRG severity, as this measure may capture acute organ dysfunction arising during hospitalization and lead to overadjustment. Second, the analysis was restricted to elective admissions to explicitly adjust for baseline comorbidities and mitigate potential overadjustment from APR-DRG severity. The elective-admission models excluded APR-DRG severity and instead adjusted explicitly for available admission-level factors, including diabetes mellitus, hypertension, renal disease, heart failure, chronic lung disease, pulmonary circulation disease, valvular disease, peripheral vascular disease, and coagulopathy.

All statistical tests were two-sided, with *p* < 0.05 considered statistically significant. Analyses were performed using R version 4.3.1 (R Foundation for Statistical Computing, Vienna, Austria).

## 3. Results

A total of 9323 unweighted hospitalizations met the inclusion criteria, corresponding to a national estimate of 46,610 hospitalizations after applying discharge weights. Of these, 7740 (16.6%) were classified as obese and 38,870 (83.4%) as non-obese. Complete baseline characteristics, alongside survey-weighted Standardized Mean Differences (SMDs), are summarized in Table 1. The mean age of the cohort was 57.9 ± 18.9 years, and 67.5% of the hospitalizations involved females. Public insurance coverage accounted for 57.7% of cases. Socioeconomic status, measured using ZIP code income quartile, was evenly distributed, with 29.2% of hospitalizations originating from individuals residing in the highest-income quartile. Clinical severity differed between groups, with obese hospitalizations exhibiting higher proportions of APR-DRG severity levels 2 and 3.

In unadjusted analyses, obese hospitalizations had a higher rate of total in-hospital complications than non-obese hospitalizations (24.9% vs. 19.1%; *p* < 0.001). As detailed in Table 2, obesity was also associated with significantly higher unadjusted rates of pulmonary, cardiac, renal, and VTE complications, as well as longer mean hospital lengths of stay. No significant unadjusted differences were observed for sepsis or total hospital charges between the groups.

**Figure 2:**
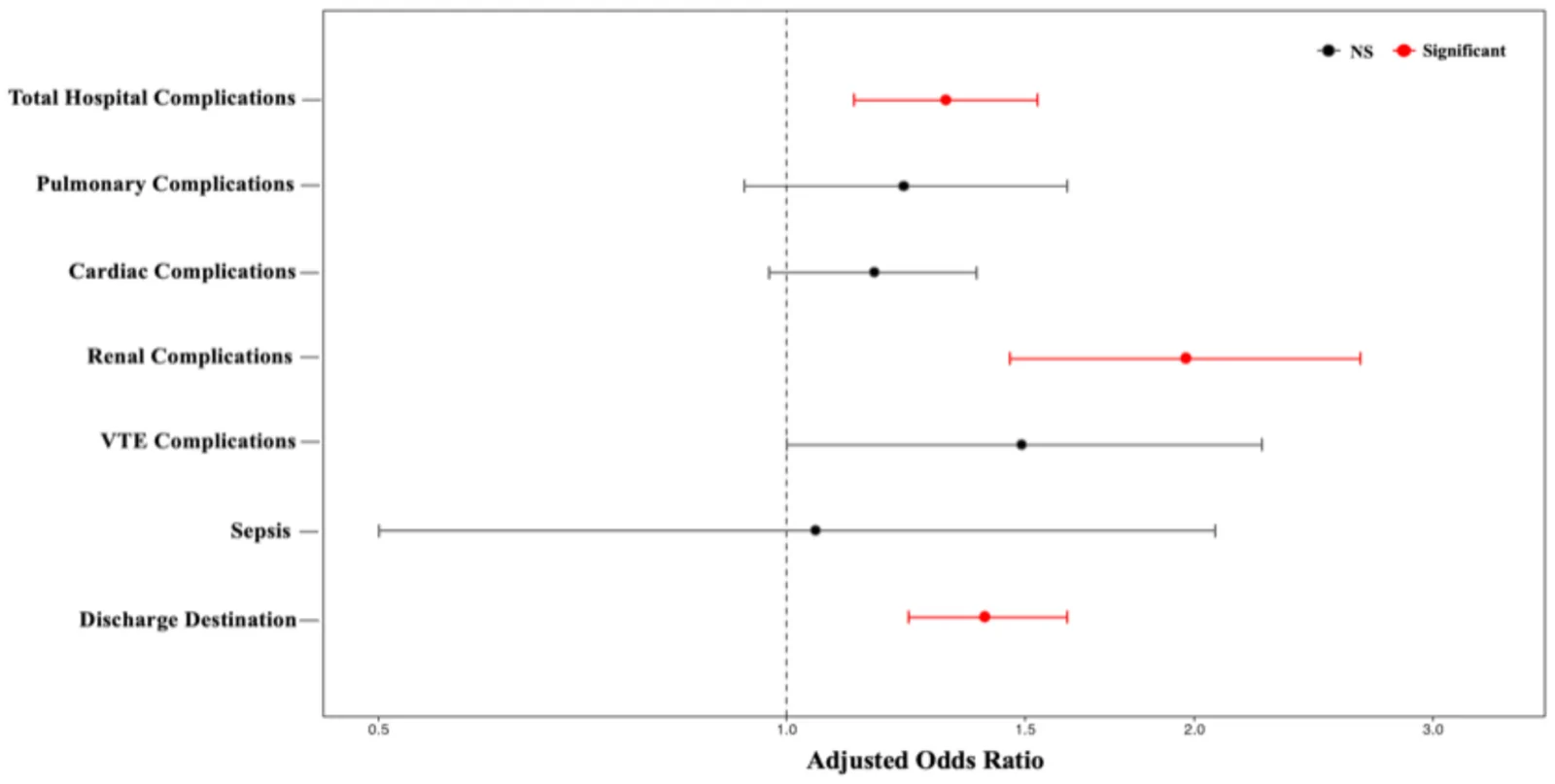
Adjusted Association of Obesity with In-Hospital Outcomes (Survey-Weighted Multivariable Analysis).

After adjustment for patient- and hospital-level covariates using survey-weighted regression models, obesity remained associated with in-hospital complications. Obesity was associated with a 22% increase in the odds of total in-hospital complications (OR, 1.22; 95% CI, 1.04–1.43; *p* = 0.02). Among individual complication categories, only in-hospital renal complications remained statistically significant (OR, 1.69; 95% CI, 1.29–2.21; *p* < 0.001), whereas associations with pulmonary, cardiac, VTE, and sepsis outcomes did not reach statistical significance (Table 3 and Figure 2). Obesity was also associated with increased odds of non-home discharge (OR, 1.28; 95% CI, 1.12–1.46; *p* < 0.001). For continuous outcomes, obesity was not significantly associated with length of stay (adjusted Incidence Rate Ratio [aIRR], 1.04; 95% CI, 1.00–1.09; *p* = 0.06) or total hospital charges (adjusted Mean Ratio [aMR], 0.97; 95% CI, 0.92–1.01; *p* = 0.136).

**Figure 3:**
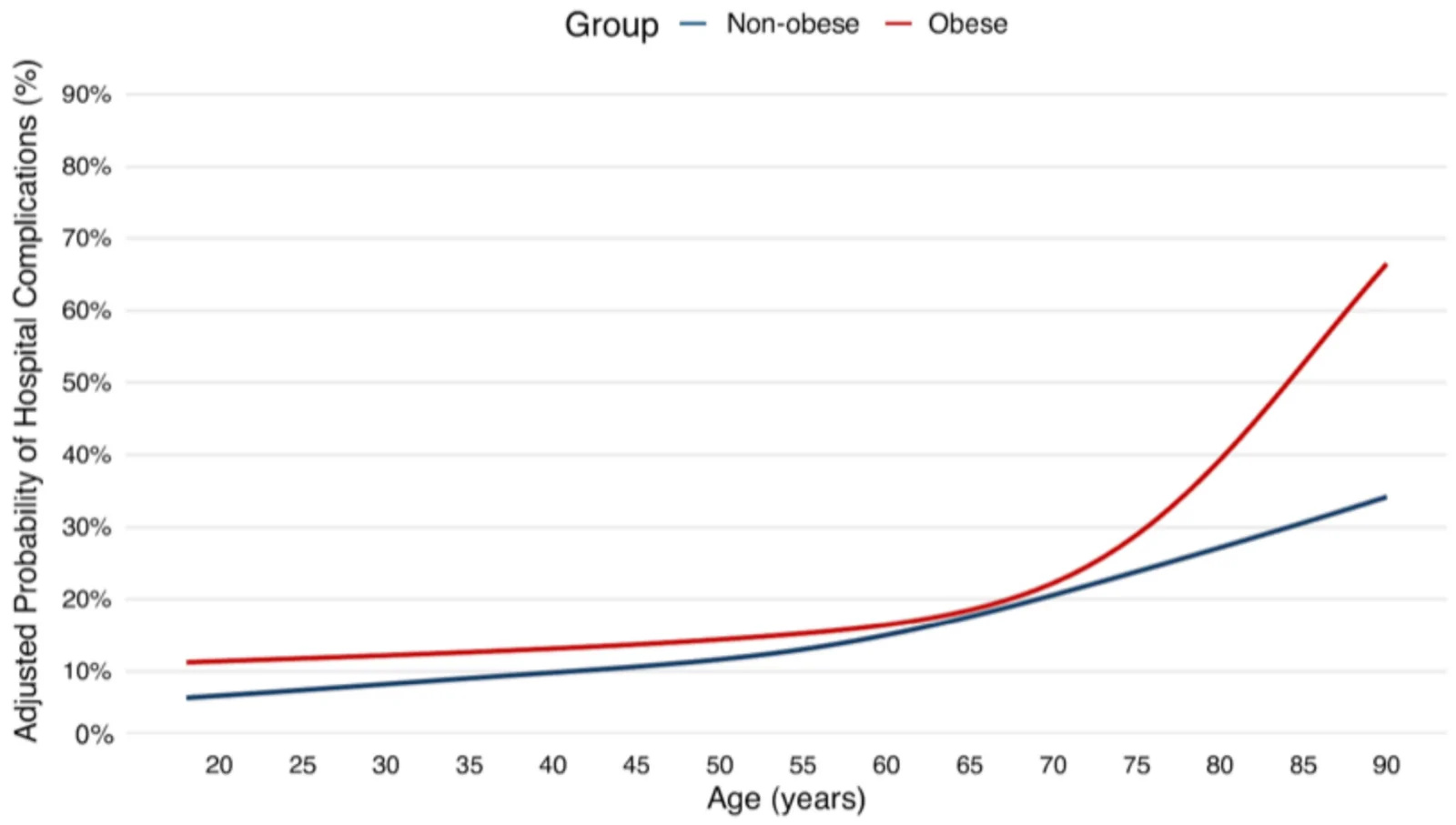
Adjusted Probability of In-Hospital Complications by Age and Obesity Status.

Moreover, the adjusted probability of in-hospital complications increased progressively with age in both groups. A formal design-adjusted joint Wald F test of all obesity-by-spline terms revealed that the overall obesity-by-age interaction was not statistically significant (*p* = 0.478). Adjusted probabilities with 95% pointwise confidence intervals are presented in Figure 3.

### Sensitivity Analyses

In the primary sensitivity analysis excluding APR-DRG severity from the adjusted models, obesity remained associated with increased odds of total in-hospital complications (OR, 1.36; 95% CI, 1.18–1.55; *p* < 0.001). Significant associations were also observed for in-hospital renal (OR, 1.83; 95% CI, 1.42–2.36; *p* < 0.001), VTE (OR, 1.57; 95% CI, 1.11–2.21; *p* = 0.01), pulmonary (OR, 1.32; 95% CI, 1.07–1.61; *p* = 0.01), and cardiac complications (OR, 1.20; 95% CI, 1.01–1.43; *p* = 0.04). No significant association was observed for sepsis or total hospital charges. Obesity was also associated with a 10% longer hospital stay (aIRR, 1.10; 95% CI, 1.05–1.16; *p* < 0.001) and increased odds of non-home discharge (OR, 1.41; 95% CI, 1.25–1.60; *p* < 0.001). Total hospital charges did not differ significantly between the groups (aMR, 1.01; 95% CI, 0.96–1.06; *p* = 0.62) (Table 4).

In the complementary sensitivity analysis restricted strictly to elective admissions and explicitly adjusting for baseline comorbidities, obesity remained associated with increased odds of total in-hospital complications (OR, 1.23; 95% CI, 1.05–1.44; *p* = 0.01), renal complications (OR, 1.55; 95% CI, 1.18–2.03; *p* = 0.002), VTE complications (OR, 1.65; 95% CI, 1.13–2.41; *p* = 0.01), and non-home discharge (OR, 1.27; 95% CI, 1.11–1.45; *p* < 0.001). Obesity was also associated with a 9% longer hospital stay (aIRR, 1.09; 95% CI, 1.04–1.15; *p* = 0.001), whereas total hospital charges did not differ significantly between the groups (Table 5).

## 4. Discussion

In this large, nationally representative study of 46,610 weighted US hospitalizations involving adults undergoing surgical correction for scoliosis, obesity was associated with increased odds of overall in-hospital complications. The strongest association was observed for renal complications. Obesity was also associated with a higher likelihood of non-home discharge. Although the association with venous thromboembolism (VTE) was not statistically significant in the primary adjusted model, it was recapitulated in the sensitivity analyses.

These findings align with both recent and foundational studies reporting higher complication rates among obese individuals undergoing spine surgery, supporting the growing body of evidence linking adiposity to increased in-hospital morbidity in adult spinal deformity surgery [2,10,13,19,20,21,22]. The mechanisms underlying this relationship are multifactorial, likely involving systemic inflammation, impaired microvascular perfusion, and a higher prevalence of metabolic comorbidities [23,24,25,26]. Additionally, obesity is known to promote a prothrombotic state and endothelial dysfunction [22,27,28].

While obesity was associated with a 22% increase in the odds of overall in-hospital complications, this was not uniformly demonstrated across all individual complication categories in the primary model. Specifically, obesity was not significantly associated with higher rates of cardiac or pulmonary complications during the index hospitalization. These findings are consistent with several previous studies examining outcomes following adult spinal deformity surgery, such as Amin et al. [4] and Elsamadicy et al. [15]. Discrepancies between these results and other cohorts reporting higher cardiopulmonary complication rates may stem from differences in patient populations, surgical complexity, or methodological variations, such as heterogeneous complication definitions and confounding adjustment strategies [22,29].

Renal complications were the only individual category consistently associated with obesity across all adjusted models. Although relatively few studies have examined this relationship specifically in adult spinal deformity surgery, Marquardt et al. similarly reported obesity as a significant risk factor for acute kidney injury following long-segment fusion [13], whereas Amin et al. did not observe a significant association [4]. The mechanisms driving this association likely involve obesity-related alterations in renal hemodynamics [30], the release of pro-inflammatory cytokines [31], and the synergistic effects of metabolic comorbidities such as diabetes and hypertension [32], as well as abdominal fat distribution [33].

Although our primary adjusted model did not demonstrate a statistically significant association between obesity and VTE, the complementary sensitivity analyses revealed an increased risk. This trend aligns with the substantial body of literature linking obesity to VTE after spine surgery. While several reviews have identified obesity as a risk factor for deep vein thrombosis and pulmonary embolism [5,34], some studies focusing specifically on adult spinal deformity surgery have reported low VTE incidence and failed to detect a significant association [9,12,13,15]. To rigorously evaluate confounding, a sensitivity analysis excluding APR-DRG severity was performed. The finding that VTE reached statistical significance only when APR-DRG was excluded suggests that the relationship between obesity and VTE in this population may be closely tied to the overall severity of the acute illness rather than baseline adiposity alone.

Beyond specific complications, obesity was associated with a higher likelihood of non-home discharge. While the association with hospital length of stay did not reach statistical significance in our primary analysis, prior studies have reported a trend toward longer hospitalizations among obese individuals [35]. Recent meta-analyses and cohort studies suggest that the underlying mechanisms may involve postoperative mobility limitations and functional recovery rather than surgical complexity [9,11]. Furthermore, Barrie et al. identified obesity as an independent predictor of discharge to rehabilitation facilities [2]. The significantly higher rates of non-home discharge observed in our study further support that obesity-related factors contribute to increased post-acute healthcare utilization.

Previous studies have also reported increased healthcare costs associated with obesity in spine surgery [36,37]. For example, Brown et al. reported higher one-year healthcare costs among obese individuals [19], and Amin et al. found greater odds of incurring high episode-of-care costs [4]. In contrast, this study did not identify a significant association between obesity and total in-hospital charges. This suggests that the financial burden associated with obesity in this population may manifest primarily during the post-acute phase of care, a premise supported by the substantially higher likelihood of non-home discharge among obese hospitalizations.

Several limitations must be considered when interpreting these findings. First, the NIS relies on administrative ICD-10-CM codes and does not provide measured height or weight. Although BMI-specific Z68 codes were queried during data extraction, high rates of missingness precluded reliable stratification by obesity class. Consequently, the binary administrative codes used likely have limited sensitivity, potentially capturing only clinically documented or severe obesity, thereby underestimating true prevalence and introducing measurement bias [38]. Second, administrative databases lack a universal present-on-admission (POA) indicator, limiting our ability to definitively establish the exact temporal sequence of all clinical conditions; thus, associations are strictly defined as in-hospital rather than definitively postprocedural. Third, the database lacks detailed medical record confounders, including granular surgical complexity metrics (e.g., operative time, number of fused levels, specific osteotomies), specific radiographic parameters, and validated frailty indices. Furthermore, despite utilizing multivariable regression models and calculating Standardized Mean Differences to account for baseline imbalances between the groups, residual confounding arising from inherent demographic and clinical differences between the obese and non-obese cohorts cannot be completely ruled out. Fourth, follow-up is strictly limited to the index hospitalization, preventing the evaluation of longitudinal outcomes beyond discharge. Finally, formal multiplicity corrections were not applied to the secondary exploratory endpoints; therefore, the findings regarding individual organ-system complications should be interpreted cautiously as hypothesis-generating.

To address these limitations, future research should prioritize prospective, deformity-specific cohort studies. Such studies must incorporate measured continuous BMI, distinct obesity classes, validated frailty indices, and detailed radiographic and operative complexity metrics to better delineate risk. Additionally, utilizing rigorous cohort matching techniques, such as propensity score matching, would help isolate the specific association of obesity by minimizing baseline demographic and clinical discrepancies. Furthermore, longitudinal tracking utilizing patient-reported outcome measures (PROMs) is essential to fully understand the long-term clinical and economic implications of obesity in adult spinal deformity surgery.

## 5. Conclusions

In this large national analysis, obesity was associated with increased in-hospital morbidity among adults undergoing surgical correction for scoliosis, particularly with respect to renal complications and a higher likelihood of non-home discharge. These population-level findings serve as a complementary epidemiological addition to the existing literature, highlighting the unique operative burden of adult spinal deformity. By quantifying these specific in-hospital risks, this study provides actionable data to optimize preoperative risk communication and to better anticipate post-acute discharge resource requirements for this complex surgical population.

## Figures and Tables

**Table 1:** Baseline characteristics stratified by obesity comorbidity.

	Non-Obese (*N* = 38,870)	Obese (*N* = 7740)	Overall (*N* = 46,610)	SMD
**Age** (years)				
Mean (SD)	57.2 (19.7)	61.6 (13.4)	57.9 (18.9)	0.26
**Sex** *n* (%)				
Male	12,795 (32.9%)	2345 (30.3%)	15,140 (32.5%)	0.06
Female	26,075 (67.1%)	5395 (69.7%)	31,470 (67.5%)	
Missing	5 (0.0%)	0 (0.0%)	5 (0.0%)	
**Race** *n* (%)				
White	31,880 (85.5%)	6535 (87.0%)	38,415 (85.9%)	0.09
Black	1960 (5.3%)	460 (6.1%)	2420 (5.4%)	
Other	3435 (9.2%)	520 (6.9%)	3955 (8.7%)	
Missing	1595 (4.1%)	225 (2.9%)	1820 (3.9%)	
**Insurance** *n* (%)				
Public	22,355 (57.5%)	4520 (58.4%)	26,875 (57.7%)	0.05
Private	15,060 (38.7%)	2975 (38.4%)	18,035 (38.7%)	
Self-pay	260 (0.7%)	25 (0.3%)	285 (0.6%)	
Other	1200 (3.1%)	220 (2.8%)	1420 (3.0%)	
**Socioeconomic Status Quartile** *n* (%)				
Q1	7070 (18.4%)	1515 (19.7%)	8585 (18.4%)	0.09
Q2	9345 (24.4%)	2120 (27.6%)	11,465 (24.6%)	
Q3	10,390 (27.1%)	1950 (25.4%)	12,340 (26.5%)	
Q4	11,525 (30.1%)	2095 (27.3%)	13,620 (29.2%)	
Missing	540 (1.4%)	62 (0.8%)	602 (1.3%)	
**APRDRG_Severity** *n* (%)				
1	15,770 (40.6%)	2055 (26.6%)	17,825 (38.2%)	0.30
2	14,700 (37.8%)	3610 (46.6%)	18,310 (39.3%)	
3	7000 (18.0%)	1790 (23.1%)	8790 (18.9%)	
4	1405 (3.6%)	285 (3.7%)	1690 (3.6%)	
**Comorbidities**				
Diabetes	4115 (10.6%)	1880 (24.3%)	5995 (12.9%)	0.37
Hypertension	18,535 (47.7%)	5400 (69.8%)	23,935 (51.4%)	0.46
Renal disease	1785 (4.6%)	610 (7.9%)	2395 (5.1%)	0.14
Heart failure	1120 (2.9%)	350 (4.5%)	1470 (3.2%)	0.09
Chronic lung disease	6910 (17.8%)	1720 (22.2%)	8630 (18.5%)	0.11
Pulmonary circulation disease	425 (1.1%)	95 (1.2%)	520 (1.1%)	0.01
Valvular disease	1425 (3.7%)	290 (3.7%)	1715 (3.7%)	0.004
Peripheral vascular disease	1145 (2.9%)	320 (4.1%)	1465 (3.1%)	0.06
Coagulopathy	3295 (8.5%)	705 (9.1%)	4000 (8.6%)	0.02
**Hospital Size** *n* (%)				
Small	8945 (23.0%)	1650 (21.3%)	10,595 (22.7%)	0.05
Medium	7960 (20.5%)	1710 (22.1%)	9670 (20.7%)	
Large	21,970 (56.5%)	4380 (56.6%)	26,350 (56.5%)	
**Year** *n* (%)				
2016	7770 (20.0%)	1185 (15.3%)	8955 (19.2%)	0.15
2017	7510 (19.3%)	1450 (18.7%)	8960 (19.2%)	
2018	6880 (17.7%)	1530 (19.8%)	8410 (18.0%)	
2019	6450 (16.6%)	1230 (15.9%)	7680 (16.5%)	
2020	5385 (13.9%)	1110 (14.3%)	6495 (13.9%)	
2021	4880 (12.6%)	1235 (16.0%)	6115 (13.1%)	

Abbreviations: APRDRG, all patient refined diagnosis related group; SD, standard deviation.

**Table 2:** Unadjusted hospitalization and in-hospital complication outcomes.

	Non-Obese (*N* = 38,870)	Obese (*N* = 7740)	Overall (*N* = 46,610)	*p*-Value
**In-Hospital Complications *n* (%)**				
Total Hospital Complications	7420 (19.1%)	1930 (24.9%)	9350 (20.1%)	<0.001
Pulmonary Complications	2750 (7.1%)	680 (8.8%)	3430 (7.4%)	0.021
Cardiac Complications	4330 (11.1%)	1050 (13.6%)	5380 (11.5%)	0.007
Renal Complications	1295 (3.3%)	485 (6.3%)	1780 (3.8%)	<0.001
VTE Complications	750 (1.9%)	230 (3.0%)	980 (2.1%)	0.012
Sepsis	305 (0.8%)	60 (0.8%)	365 (0.8%)	0.97
**Hospitalization Outcomes**				
Length of Stay (Days)	5.77 (6.10)	6.29 (5.32)	5.85 (5.98)	<0.001
Total Charges (USD)	268,000 (205,000)	273,000 (204,000)	269,000 (205,000)	0.46
**Discharge Destination**				
Non-Home	11,975 (30.8%)	3070 (39.7%)	15,045 (32.3%)	<0.001
Home	26,820 (69.0%)	4665 (60.3%)	31,485 (67.5%)	
Missing	75 (0.2%)	8 (0.1%)	83 (0.2%)	

**Table 3:** Survey-weighted adjusted associations between obesity and in-hospital outcomes.

Categorical Outcomes	Adjusted Estimate	Confidence Interval	*p*-Value
Total Hospital Complications	1.22	1.04 to 1.43	0.02
Pulmonary Complications	1.18	0.91 to 1.52	0.22
Cardiac Complications	1.08	0.90 to 1.30	0.39
Renal Complications	1.69	1.29 to 2.21	<0.001
VTE Complications	1.34	0.93 to 1.92	0.11
Sepsis	1.05	0.51 to 2.14	0.90
Discharge Destination (Non-Home)	1.28	1.12 to 1.46	<0.001
**Continuous Outcomes**	**Adjusted Estimate**	**Confidence Interval**	***p*-Value**
Length of Stay (Days)	1.04	1.00 to 1.09	0.06
Total Charges (USD)	0.97	0.92 to 1.01	0.136

Abbreviations: VTE, venous thromboembolism; USD, United States dollars.

**Table 4:** Survey-weighted adjusted associations between obesity and in-hospital outcomes excluding APRDRG severity (sensitivity analysis).

Categorical Outcomes	Odds Ratio	Confidence Interval	*p*-Value
Total Hospital Complications	1.36	1.18 to 1.55	<0.001
Pulmonary Complications	1.32	1.07 to 1.61	0.01
Cardiac Complications	1.20	1.01 to 1.43	0.04
Renal Complications	1.83	1.42 to 2.36	<0.001
VTE Complications	1.57	1.11 to 2.21	0.01
Sepsis	1.18	0.62 to 2.24	0.62
Discharge Destination (Non-Home)	1.41	1.25 to 1.60	<0.001
**Continuous Outcomes**	**Adjusted Estimate**	**Confidence Interval**	***p*-Value**
Length of Stay (Days)	1.10	1.05 to 1.16	<0.001
Total Charges (USD)	1.01	0.96 to 1.06	0.62

Abbreviations: VTE, venous thromboembolism; USD, United States dollars.

**Table 5:** Survey-weighted adjusted associations between obesity and in-hospital outcomes among elective admissions, with explicit comorbidity adjustment (sensitivity analysis).

Categorical Outcomes	Odds Ratio	Confidence Interval	*p*-Value
Total Hospital Complications	1.23	1.05 to 1.44	0.01
Pulmonary Complications	1.22	0.98 to 1.52	0.07
Cardiac Complications	1.05	0.85 to 1.29	0.67
Renal Complications	1.55	1.18 to 2.03	0.002
VTE Complications	1.65	1.13 to 2.41	0.01
Sepsis	1.12	0.57 to 2.20	0.75
Discharge Destination (Non-Home)	1.27	1.11 to 1.45	<0.001
**Continuous Outcomes**	**Mean Difference**	**Confidence Interval**	***p*-Value**
Length of Stay (Days)	1.09	1.04 to 1.15	0.001
Total Charges (USD)	1.00	0.95 to 1.05	0.966

Abbreviations: VTE, venous thromboembolism; USD, United States dollars.

## Data Availability

These data were derived from the following public-domain resource: the Healthcare Cost and Utilization Project (HCUP) National Inpatient Sample (NIS) database at https://cdors.ahrq.gov/databases (accessed on 14 June 2026).

## Supplementary Materials

The following supporting information can be downloaded at https://www.mdpi.com/article/10.3390/jcm15176511/s1, Supplemental Table S1. ICD-10 Diagnosis and Procedure Codes Used for Cohort Identification, Baseline Comorbidities, and In-Hospital Outcomes; Supplemental Table S2. STROBE Statement checklist of items that should be included in reports of observational studies.
