# Supplementary material for: Quantifying the Association Between Obesity and In-Hospital Outcomes in Adult Scoliosis Surgery: A Nationwide Inpatient Sample Analysis (2016–2021)
Source: J Clin Med. 2026 Aug 23;15(17):6511. doi: 10.3390/jcm15176511 (PMC13566887; doi:10.3390/jcm15176511)
Supplement: Supplementary file 1 [file jcm-15-06511-s001.zip › jcm-4433787-supplementary.pdf]

Supplemental Table S1. ICD-10 Diagnosis and Procedure Codes Used for Cohort Identification, Baseline Comorbidities, and In-Hospital Outcomes.

Category	Code type	Codes
**Cohort Identification**		
Scoliosis Diagnosis	ICD-10-CM	M4102, M4103, M41112, M41113, M41122, M41123, M4122, M4123, M4132, M4133, M4142, M4143, M4152, M4153, M4182, M4183, M4104, M4105, M41114, M41115, M41124, M41125, M4124, M4125, M4134, M4135, M4144, M4145, M4154, M4155, M4184, M4185, M4106, M4107, M41116, M41117, M41126, M41127, M4126, M4127, M4136, M4137, M4146, M4147, M4156, M4157, M4186, M4187, M4108, M4109, M41118, M41119, M41128, M41129, M4128, M4129, M4138, M4139, M4148, M4149, M4158, M4159, M4188, M4189
Scoliosis correction	ICD-10-PCS	0RG7070, 0RG7071, 0RG707J, 0RG70A0, 0RG70A1, 0RG70AJ, 0RG70J0, 0RG70J1, 0RG70JJ, 0RG70K0, 0RG70K1, 0RG70KJ, 0RG70Z0, 0RG70Z1, 0RG70ZJ, 0RG7370, 0RG7371, 0RG73K1, 0RG73Z1, 0RG7470, 0RG7471, 0RG747J, 0RG74A0, 0RG74A1, 0RG74J0, 0RG74J1, 0RG74K0, 0RG74K1,

Category	Code type	Codes
		0RG74Z0, 0RG74Z1, 0RG8070, 0RG8071, 0RG807J, 0RG80A0, 0RG80A1, 0RG80AJ, 0RG80J1, 0RG80JJ, 0RG80K0, 0RG80K1, 0RG80KJ, 0RG80Z1, 0RG80ZJ, 0RG8371, 0RG83A1, 0RG83Z1, 0RG8470, 0RG8471, 0RG84J0, 0RG84K1
**Baseline Comorbidities (Adjusted in Sensitivity Models)**		
Diabetes Mellitus	ICD-10-CM	E11
Hypertension	ICD-10-CM	I10
Renal Disease	ICD-10-CM	N18
Heart Failure	ICD-10-CM	I500, I501, I5021, I5031, I5033, I5041, I5043, I509
Chronic Lung Disease	ICD-10-CM	J44
Pulmonary Circulation Disease	ICD-10-CM	I27
Valvular Disease	ICD-10-CM	I34, I35
Peripheral Vascular Disease	ICD-10-CM	I73
Coagulopathy	ICD-10-CM	D68

Category	Code type	Codes
**In-Hospital Postprocedural Complications (Outcomes)**		
**Pulmonary complications** (includes pneumonia, ARDS, acute respiratory failure, other postprocedural respiratory disorders)	ICD-10-CM	J12, J13, J14, J15, J16, J17, J18, J22, J80, J96, A481, J810, J952, J953, J954, J956, J958, J959, J984, J9572, J9801, J95811, J95812, J95821, J95822, J95850, J95851, J95859
**Cardiac complications** (includes acute myocardial infarction, cardiac arrest, cardiogenic shock, postprocedural heart failure)	ICD-10-CM	I20, I21, I22, I23, I24, I46, I971, T811, I9788, I9789, I97111, I97121, I97131, I97191, I97711, I97791
**Renal complications** (includes acute kidney failure, UTI, other postprocedural renal disorders)	ICD-10-CM	N10, N17, N19, N30, R33, N390, N990, N9981, N9989
**Venous thromboembolism**	ICD-10-CM	I26, I80, I81, I82, T8171, T8172

Category	Code type	Codes
(includes DVT and pulmonary embolism)		
**Sepsis**	ICD-10-CM	A40, A41, R652, T8144

Supplemental Table S2. STROBE Statement checklist of items that should be included in reports of observational studies

Section / Topic	Item No	Recommendation	Reported on Section / Paragraph
**Title and abstract**			
	1 (a)	Indicate the study's design with a commonly used term in the title or the abstract.	Title; Abstract (Methods)
	1 (b)	Provide in the abstract an informative and balanced summary of what was done and what was found.	Abstract
**Introduction**			
Background/rationale	2	Explain the scientific background and rationale for the investigation being reported.	Introduction, Paragraphs 1–2
Objectives	3	State specific objectives, including any prespecified hypotheses.	Introduction, Final Paragraph
**Methods**			
Study design	4	Present key elements of study design early in the paper.	Methods: Study Design and Data Source

Section / Topic	Item No	Recommendation	Reported on Section / Paragraph
Setting	5	Describe the setting, locations, and relevant dates, including periods of recruitment, exposure, follow-up, and data collection.	Methods: Study Design and Data Source (NIS 2016–2021)
Participants	6 (a)	Cohort study: Give the eligibility criteria, and the sources and methods of selection of participants. Describe methods of follow-up.	Methods: Study Population; Figure S1
Variables	7	Clearly define all outcomes, exposures, predictors, potential confounders, and effect modifiers. Give diagnostic criteria, if applicable.	Methods: Exposure, Outcomes, Covariates; Supplemental Table 1
Data sources/ measurement	8	For each variable of interest, give sources of data and details of methods of assessment (measurement).	Methods: Study Design, Exposure, Outcomes; Supplemental Table 1
Bias	9	Describe any efforts to address potential sources of bias.	Methods: Sensitivity Analysis (addressing overadjustment and indication bias)

Section / Topic	Item No	Recommendation	Reported on Section / Paragraph
Study size	10	Explain how the study size was arrived at.	Methods: Study Population (All eligible discharges)
Quantitative variables	11	Explain how quantitative variables were handled in the analyses. If applicable, describe which groupings were chosen and why.	Methods: Statistical Analysis (Splines for age; modeling of skewed continuous variables)
Statistical methods	12 (a)	Describe all statistical methods, including those used to control for confounding.	Methods: Statistical Analysis
	12 (b)	Describe any methods used to examine subgroups and interactions.	Methods: Statistical Analysis (Obesity-by-age interaction)
	12 (c)	Explain how missing data were addressed.	Methods: Missing Data
	12 (d)	Cohort study: If applicable, explain how loss to follow-up was addressed.	N/A (In-hospital database; no longitudinal follow-up)
	12 (e)	Describe any sensitivity analyses.	Methods: Sensitivity Analysis
**Results**			

Section / Topic	Item No	Recommendation	Reported on Section / Paragraph
Participants	13 (a)	Report numbers of individuals at each stage of study (e.g., potentially eligible, included, analyzed).	Results, Paragraph 1; Figure S1
	13 (b)	Give reasons for non-participation at each stage.	Figure S1 (Exclusion criteria detailed)
	13 (c)	Consider use of a flow diagram.	Figure S1
Descriptive data	14 (a)	Give characteristics of study participants and information on exposures and potential confounders.	Results, Paragraph 1; Table 1
	14 (b)	Indicate number of participants with missing data for each variable of interest.	Table 1
Outcome data	15	Cohort study: Report numbers of outcome events or summary measures over time.	Table 2
Main results	16 (a)	Give unadjusted estimates and, if applicable, confounder-adjusted estimates and their precision (e.g., 95% CI). Make	Results, Paragraphs 3-4; Table 3; Figure 2

Section / Topic	Item No	Recommendation	Reported on Section / Paragraph
		clear which confounders were adjusted for.	
	16 (b)	Report category boundaries when continuous variables were categorized.	N/A (Modeled continuously)
	16 (c)	If relevant, consider translating estimates of relative risk into absolute risk for a meaningful time period.	Results (Predicted probabilities text); Figure 3
Other analyses	17	Report other analyses done (e.g., analyses of subgroups and interactions, and sensitivity analyses).	Results: Sensitivity Analyses; Tables 4 & 5
**Discussion**			
Key results	18	Summarise key results with reference to study objectives.	Discussion, Paragraph 1
Limitations	19	Discuss limitations of the study, taking into account sources of potential bias or imprecision. Discuss both direction and magnitude of any potential bias.	Discussion, Limitations Paragraphs

**Section / Topic**	**Item No**	**Recommendation**	**Reported on Section / Paragraph**
Interpretation	20	Give a cautious overall interpretation of results considering objectives, limitations, multiplicity of analyses, results from similar studies, and other relevant evidence.	Discussion, Paragraphs 2–6
Generalisability	21	Discuss the generalisability (external validity) of the study results.	Introduction; Discussion (Nationally representative sample emphasized)
**Other information**			
Funding	22	Give the source of funding and the role of the funders for the present study.	Funding section
